# Therapeutic Strategies for MASH: An Update on Drug Candidates Under Investigation in Late-Phase Clinical Trials

**DOI:** 10.3390/ijtm5010007

**Published:** 2025-01-17

**Authors:** Samuel Dinerman, Yan Shu

**Affiliations:** Department of Pharmaceutical Sciences, School of Pharmacy, University of Maryland at Baltimore, Baltimore, MD 21201, USA

**Keywords:** metabolic dysfunction-associated steatohepatitis, drug development, therapeutics, clinical trials

## Abstract

Metabolic dysfunction-associated steatohepatitis (MASH) is rapidly becoming a leading cause of hepatocellular carcinoma and end-stage liver transplantation. Characterized by hepatic steatosis, lobular inflammation, and hepatocyte ballooning, there is a dire need to develop therapeutic strategies to mitigate MASH alongside the subsequent fibrosis and cirrhosis. For years, therapeutic development for the treatment of MASH had been considered a graveyard, with various pharmacotherapies failing to achieve clinical efficacy. However, the recent Food and Drug Administration (FDA) approval of Madrigal Pharmaceuticals’ Resmetirom in the United States provides a positive step in the collective effort to eradicate MASH. Granted, with much about Resmetirom’s long-term efficacy and safety still to be determined and with the multi-factorial nature of MASH pathogenesis, continuing to evaluate alternative therapeutic options remains in the best interest of the field. Currently, therapeutics previously approved for other ailments, alongside novel therapeutics developed specifically for the treatment of MASH, are being evaluated in late-phase clinical trials. However, considering the complex nature of the disease and varying clinical outcomes to assess treatment efficacy, achieving regulatory approval as a MASH therapeutic continues to be a rigorous endeavor. In this review, we summarize notable therapeutics of various mechanistic backgrounds having achieved, or actively undergoing, late-phase clinical trials for the treatment of MASH and offer our perspectives on anti-MASH therapeutic development.

## 1. Introduction

Metabolic dysfunction-associated steatotic liver disease (MASLD), previously named non-alcoholic fatty liver disease (NAFLD) [1], is the most prevalent form of chronic liver disease in the world and has come to affect more than one billion individuals [2,3]. As of 2021, the socioeconomic burden of this disease has significantly risen in all age groups, with people over the age of 50 being the most vulnerable to the development of MASLD [4]. A more severe form of MASLD, metabolic dysfunction-associated steatohepatitis (MASH), or, as previously named, non-alcoholic steatohepatitis (NASH), has also seen a significant increase in prevalence by at least twofold over the past several decades [5]. As a worsening stage of MASLD, MASH is characterized by lobular inflammation and hepatocyte ballooning, leading to swift progression to liver fibrosis and eventual cirrhosis [3,6]. However, these phenomena are only manifested in its later stages, making MASH a “silent” liver disorder [6]. Symptoms of advanced-stage MASH include fatigue, weight loss, weakness, fluid retention, intestinal bleeding, and liver failure, making it the leading cause of both end-stage liver disease and liver transplantation [7]. Until Madrigal Pharmaceuticals’ very recent breakthrough in March 2024 with Resmetirom’s approval for MASH treatment in the United States (US), a selective thyroid hormone receptor-β (THR-β) agonist, there have been no approved pharmacotherapies for the treatment of MASH, forcing patients to primarily rely on general lifestyle interventions (e.g., diet, exercise) [6,8].

Various pharmacotherapeutic agents have been assessed to ameliorate these symptoms, some of which have reached Phase 3 clinical trials [6]. However, amongst this diverse group of therapies, limitations regarding their duration of treatment, endpoints, and even patient adherence have hindered their approval. Attributed to its slowly progressive nature, with onset to development taking up to several years, selecting a uniform, clinically relevant endpoint for MASH has been a recurring challenge [9]. For example, although Resmetirom shows efficacy in MASH resolution without worsening fibrosis (25.9% of patients with 80 mg and 25.9% with 100 mg treatment vs. 14.2% with placebo; *p* < 0.001, n = 955), its primary endpoints were only from one year of treatment, hindering our ability to assess the long-term toxicity potential from repeated use beyond one year [8,10]. Indeed, with Resmetirom’s approval for MASH treatment limited to the US at this time, several clinical trials are still ongoing or recruiting to further assess Resmetirom’s efficacy and safety in varying patient populations in the hopes for the therapeutic’s eventual approval in the rest of the world (NCT05500222, NCT03900429, NCT04951219). Several papers published within the past year have offered more information regarding the mechanistic background, results, and future perspective of Resmetirom’s therapeutic potential for MASH [11–13].

As of 2019, accepted endpoints for conditional approval are MASH resolution without worsening fibrosis and/or fibrosis improvement by at least one stage without worsening MASH [14]. However, these endpoints must differ based upon fibrosis stage, which is a challenge to assess itself due to the nonlinear progression of the disease [15]. Additionally, the instance of response to placebo has created issues in the past as a small portion of the placebo/control groups in several MASH clinical trials had noticeable efficacy, rendering some clinically significant interpretations of the therapeutic being evaluated meaningless [6,15]. This has been attributed to lifestyle modifications recommended by clinical trial sponsors, clouding whether the evaluated histological features of MASH in the trial are only a consequence of the experimental therapeutic in question [15]. Finally, there remains a lack of inter- and intra-reader objectivity in the evaluation of treatment efficacy at the end of clinical trials, which has resulted in calls for establishing universal guidelines for patient evaluation and pathological diagnosis at the end of treatment periods [15–18].

Currently, the only confirmed method of assessing treatment efficacy for MASH is liver biopsy, resulting in repeated refusal from patients in these trials to undergo this invasive procedure. Furthermore, the biopsy specimens only represent a small portion of the entire organ, and the procedure can cause complications in the patients, including bleeding and even mortality [19,20]. Several non-invasive tests (NITs) have shown promise in their assessment of MASH progression and/or its treatment outcome [21]. Based on the recommendations of the American Gastroenterological Association (AGA) and American Association for the Study of Liver Diseases (AASLD), both blood-based and imaging-based NITs can be utilized in lieu of biopsy for the baseline evaluation of MASH in clinical settings [22–24]. However, there remain several hurdles to the universal integration of NITs in favor of biopsy. There remains insufficient data to recommend the use of any NITs alone for longitudinal assessment [24]. As a result, for the purpose of risk stratification, several sponsors have resorted to using NITs with paired biopsy for not only confirming the diagnosis of MASH but also assessing the predictive value of these NITs for the eventual replacement of histological assessment. Other hurdles to NIT implementation are concerned with the general availability, cost-effectiveness, and healthcare provider expertise, especially with imaging-based NITs [22,24]. Finally, there remains inconsistency in the ability of NITs to discern MASH from simple steatosis, along with detecting the advancement of hepatic fibrosis [21].

Beyond those already advanced to clinical trials, a plethora of agents and potential therapeutic targets are currently being evaluated in preclinical and early clinical development. However, difficulties remain in determining the most efficacious manner to target MASH, due to its implications in not only liver function, but other systemic functions throughout the body, ranging from glucose metabolism to immune response. In this review, we summarize current pharmacotherapeutic strategies that have reached late-phase clinical trials (i.e. Phases 2b, 3) and their major mechanisms of action to target and alleviate the symptoms of MASH.

## 2. Promising MASH Therapeutic Agents in Late-Phase Clinical Trials and Their Targets

A plethora of pathways have been implicated in the pathology of MASH development, involving specific receptors, signaling cascades, and metabolic enzymes. These pathways have been implicated in the regulation and manipulation of MASH pathologies, ranging anywhere from cellular stress and immune responses to tissue regeneration and fibrosis pathogenesis [25]. Amongst the numerous pathways or molecular targets being evaluated, a select few have been of high priority in the pharmaceutical world’s quest to target and ameliorate MASH, with drug candidates already advanced to late-phase clinical trials (Table 1).

### 2.1. GLP-1R Agonists

Glucagon-like peptide-1 (GLP-1) is an incretin hormone formed from proglucagon and predominantly produced in the intestinal epithelium [35]. Through nutrient-dependent secretion, GLP-1 stimulates insulin secretion while inhibiting glucagon release and gastric emptying; therefore, inhibiting postprandial glucose increases. GLP-1 exhibits these effects through interactions with its receptor, GLP-1R [36]. Primarily expressed in the β-cells of the Islets of Langerhans in the pancreas, ligand-bound GLP-1Rs engage with the guanine nucleotide-binding α stimulatory subunit (Gα_s_) of the heterotrimeric G protein complex, activating adenylate cyclase to generate cyclic adenosine monophosphate (cAMP) [37,38]. Increased levels of cAMP allow for calcium influx through interaction with protein kinase A (PKA), increasing insulin vesicle competency for calcium-dependent exocytosis [39].

**Figure 1.**
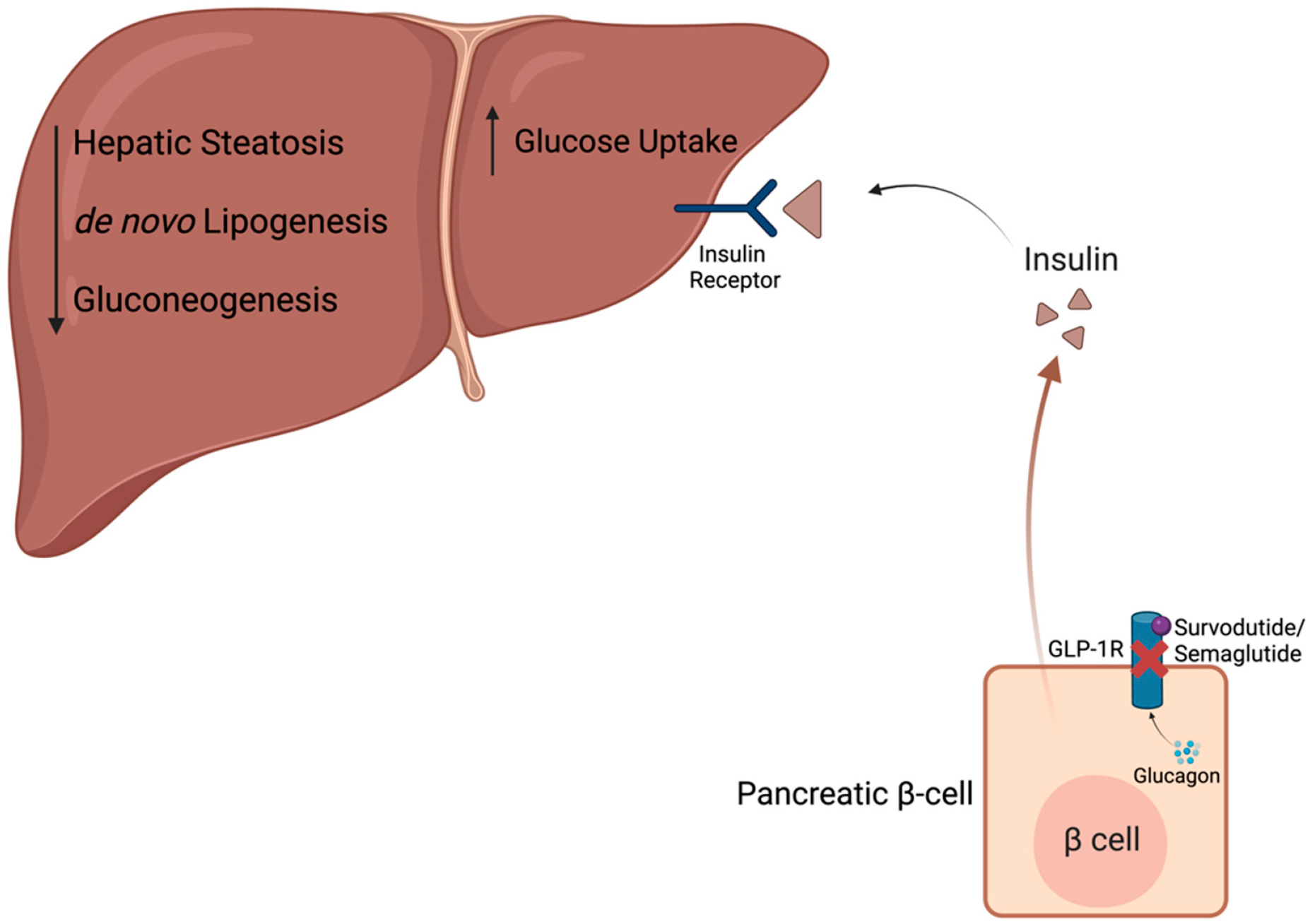
Mechanism of action for GLP-1R agonists. Primarily expressed in the Islets of Langerhans in pancreatic β-cells, glucagon-like peptide-1 receptor (GLP-1R) is bound and activated by semaglutide/survodutide, inducing insulin secretion while inhibiting glucagon release. As insulin binds to hepatic insulin receptors, glucose uptake increases, resulting in decreased hepatic steatosis, de novo lipogenesis, and gluconeogenesis. Created in BioRender.

In the liver, due to GLP-1-induced insulin secretion from the pancreas into the liver (Figure 1), hepatic insulin receptor activity increases, thereby inhibiting hepatic glucose production and de novo lipogenesis while increasing hepatic glucose uptake [40,41]. Additionally, GLP-1R activation reduces circulating levels of very low-density lipoproteins (VLDL), which transport lipids from the liver to extrahepatic tissues [40]. Due to these findings, GLP-1R agonists (GLP-1RA) have been extensively studied as possible treatments for MASLD and MASH. Through preclinical and clinical studies, researchers determined that postprandial levels of GLP-1 were decreased in MASLD and MASH patients (8.8 ± 0.7 pmol/L in patients vs. 11.7 ± 1.0 pmol/L in healthy controls; *p* < 0.001), while GLP-1RA treatment in mice was able to reduce hepatic steatosis [42–44]. GLP-1RAs have also been shown to reduce both systemic and hepatic inflammation in both MASH and non-MASH settings; however, the mechanisms by which this is achieved have yet to be determined [40]. Notably, the majority of the findings regarding GLP-1RAs’ impact on inflammation is confounded by the simultaneous improvement of various metabolic parameters in these studies [40].

Nevertheless, in a Phase 2 trial, it was determined that, compared to the placebo, semaglutide treatment (0.1, 0.2, or 0.4 mg/day) for 72 weeks resulted in a significantly higher percentage of patients (n = 320) achieving MASH resolution (40% of patients with 0.1 mg, 36% with 0.2 mg, and 59% with 0.4 mg treatment vs. 17% with placebo; *p* < 0.001 for 0.4 mg treatment vs. placebo) [45]. However, there was no significant improvement in fibrosis score in these patients with fibrosis stages from F1 to F3 (43% of patients with 0.4 mg treatment vs. 33% with placebo; *p* = 0.48). In addition, there was a significant number of patients receiving the highest dose of 0.4 mg semaglutide daily who had to stop treatment due to gastrointestinal disorders, such as nausea (42% with treatment vs. 11% with placebo), constipation (22% with treatment vs. 12% with placebo), and vomiting (15% with treatment vs. 2% with placebo) [45].

For the clinical trial “Effect of Semaglutide in Subjects with Non-cirrhotic Nonalcoholic Steatohepatitis (ESSENCE)” (NCT04822181), researchers are recruiting MASH patients (n = 1200) with a fibrosis stage of either 2 or 3, according to criteria provided by NASH Clinical Research Network (NASH CRN), along with an NAFLD activity score (NAS) of at least 4, based on the assessment of three aspects of the liver histology (steatosis, lobular inflammation, and hepatocyte ballooning), to self-administer semaglutide once per week for 72 weeks [46]. The researchers will assess semaglutide’s efficacy based on MASH resolution and fibrosis regression. Then, pending the results of the 72-week study, the researchers will continue treatment for roughly 5 years to assess cirrhosis-free survival as a measure of long-term efficacy.

A separate sponsor is also looking into survodutide’s (Figure 1) efficacy in two Phase 3 trials of obesity patients with presumed MASH (NCT06309992) or biopsy-confirmed MASH with moderate to severe fibrosis (NCT06632444). In a recently completed Phase 2 trial of MASH patients (n = 293), 2.4 mg survodutide treatment once per week proved to significantly improve MASH without worsening fibrosis (47% of patients with treatment vs. 14% with placebo; *p* < 0.001), providing the rationale for continued investigation in a Phase 3 clinical trial [47]. The Phase 3 trial studying obese patients with presumed MASH (NCT06309992) will assess survodutide’s efficacy in liver fat reduction, measured by magnetic resonance imaging proton density fat fraction (MRI-PDFF) and changes in body weight after 48 weeks of treatment. Alternatively, the LIVERAGE^™^ Phase 3 trial amongst biopsy-confirmed MASH patients, according to the same diagnostic criteria detailed in the ESSENCE clinical trial (NCT06632444), will assess the compound’s efficacy based on MASH resolution and fibrosis regression after one year of treatment. Then, pending the results after one year of treatment, the patients will continue survodutide treatment for up to seven years to assess cirrhosis-free survival as a measure of long-term efficacy.

The major hindrance to the approval of GLP-1 agonists as MASH therapeutics might be the current unrepresentative patient populations for MASH and MASLD in these clinical trials [26]. Considering that GLP-1 agonists have initially been approved to treat obese and diabetic patients, sponsors have been careful to ensure that their participants in their trials met one, or both, of these criteria. However, the ESSENCE and LIVERAGE^™^ trials will serve to be a useful barometer for the efficacy of a GLP-1 agonist in both obese and non-obese MASH patients without Type 2 Diabetes (T2D).

### 2.2. FGF21 Analogues

Fibroblast growth factor 21 (FGF21) is a signaling protein belonging to a subfamily of endocrine FGFs that regulate metabolism [48,49]. FGF21 is specifically responsible for the regulation of glucose and lipid homeostasis along with energy metabolism [50]. Unlike non-endocrine FGFs, which require heparan sulfate as a coreceptor for FGF receptor (FGFR) activation, endocrine FGFs, such as FGF21, lack this heparin-binding domain to instead form a cell surface receptor complex with FGFR and β-Klotho (KLB) coreceptor on hepatic stellate cells (HSCs) [27,51]. Through this, FGF21 inhibits HSC activation into myofibroblasts to produce extracellular matrix proteins, such as collagen, required for fibrogenesis upon liver injury [27,52,53].

The liver serves as the major source of serum FGF21 in the body [54]. Hepatic FGF21 levels can be induced in both nutrient deficiency (e.g., starvation) and excess (e.g., simple sugars) [48]. FGF21 regulates lipid and free fatty acid metabolism in the liver to protect against nutrient-stress-induced steatosis while simultaneously increasing glucose uptake in adipocytes [48,55]. In MASLD/MASH settings, FGF21 serves to reduce hepatocyte stress and hepatic steatosis, protecting against the inflammatory and fibrotic processes associated with the diseases. This is accomplished through the regulation of metabolic gene expression and mitochondrial function [48]. Further, through its secretion by stressed hepatocytes, FGF21 simultaneously modulates the activity and phenotype of Kupffer cells, a hepatic subset of macrophages, shifting the macrophage phenotype away from the induction of inflammatory response in favor of a pro-repair phenotype [27].

Considering FGF21’s pathological role in MASLD/MASH, FGF21 has recently become a hot topic as a potential MASH therapeutic. The major caveat is that FGF21 has a halflife as short as 30 min due to its susceptibility to the endopeptidase-mediated cleavage of its C-terminal domain, which is required for KLB receptor binding [27,56,57]. As a result, researchers have resorted to engineering exogenous FGF21 analogues to increase the plasma half-life, and the results so far have been very promising in both preclinical and especially clinical settings [27].

**Figure 2.**
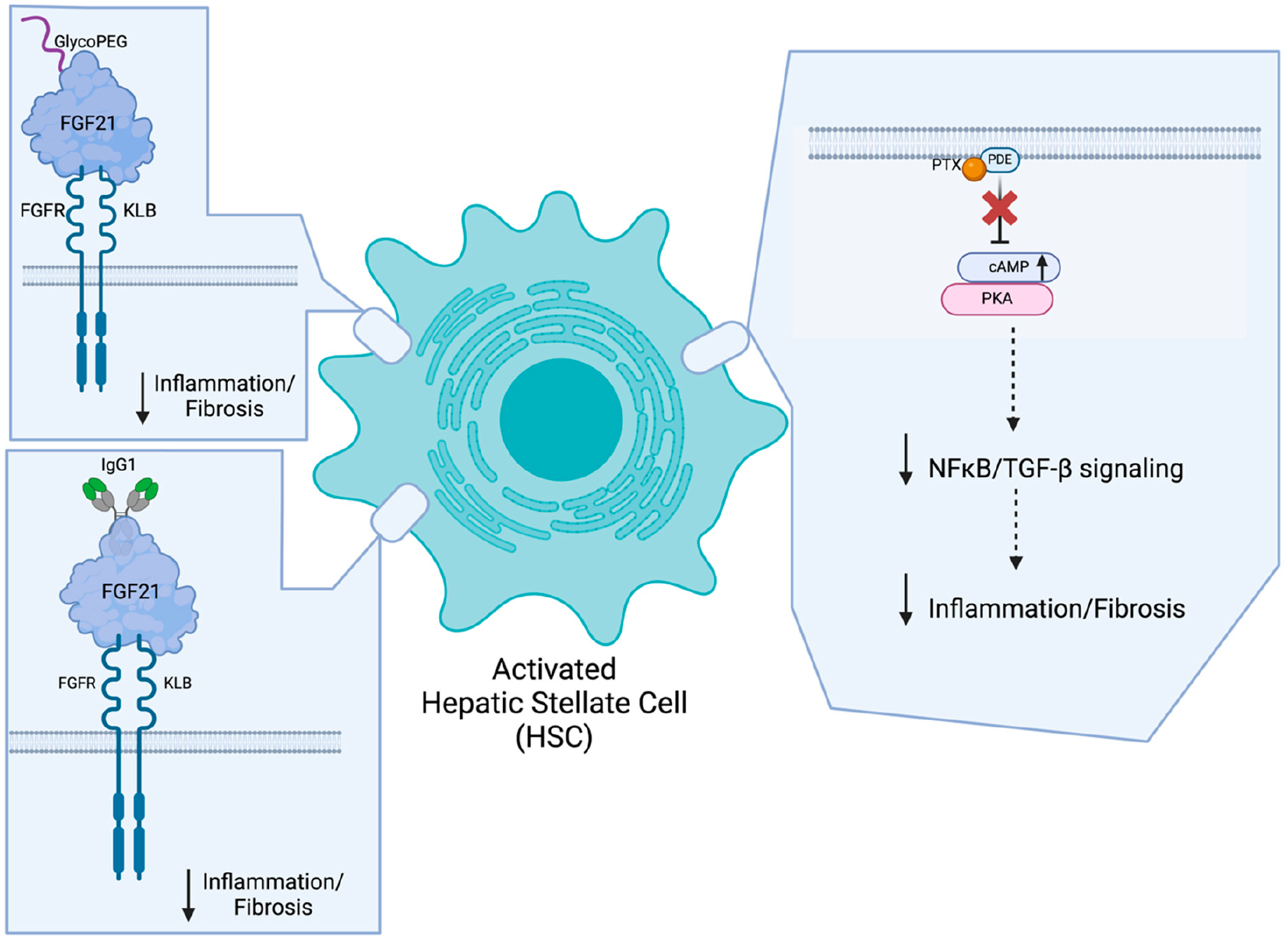
Potential MASH therapeutics primarily targeting hepatic stellate cells. Considering the instrumental role of hepatic stellate cells (HSCs) in the production of collagen and other extracellular matrix (ECM) proteins required for the development of hepatic fibrosis as it relates to MASH, developing therapeutics that directly act on this hepatic cell type has its advantages. Pentoxifylline (PTX) binds and inhibits phosphodiesterases (PDEs), resulting in elevated cyclic adenosine monophosphate (cAMP) levels required for protein kinase A (PKA) activation. Once activated, PKA suppresses nuclear factor kappa B (NF-κB) and transforming growth factor (TGF)-β-mediated signaling required for inflammatory and fibrotic processes. Efruxifermin (bottom left), a fusion protein of the human crystallized fragment (Fc) of immunoglobulin class G_1_ (IgG_1_) domain linked to a modified human FGF21 protein (Fc-FGF21), and Pegozafermin (top left), a glyco-polyethylene glycolated(glyco-PEGylated) FGF21 protein, bind to and activate fibroblast growth factor receptor (FGFR), in conjunction with β-Klotho (KLB) coreceptor, down-regulating inflammatory and fibrotic processes in hepatocytes. Created in BioRender.

Efruxifermin (Figure 2) is a fusion protein of a human crystallized fragment (Fc) of the immunoglobulin class G_1_ (IgG_1_) domain linked to a modified human FGF21 protein (Fc-FGF21) [58,59]. In a Phase 2a trial of MASH patients (n = 110), 28 mg efruxifermin treatment once per week for 12 weeks proved efficacious in significantly decreasing hepatic fat fractions, as measured by MRI-PDFF (12.1% decrease with treatment vs. 0.3% increase with placebo; *p* < 0.0001) [58]. This led the same sponsor to begin recruitment for two Phase 3 trials of efruxifermin in MASH patients with MASLD (NCT06161571) or fibrosis (NCT06215716). NCT06161571 will be more focused on elucidating the safety profile of 50 mg efruxifermin treatment for one year (n = 700), while NCT06215716 assesses the efficacy of 28 mg and 50 mg efruxifermin treatment for one year (n = 1650) to resolve MASH while improving fibrosis.

Alternatively, pegozafermin (Figure 2), a glyco-polyethylene glycolated (glyco-PEGylated) FGF21 analogue, is also making noticeable progress towards becoming a MASH therapeutic [60]. In a Phase 2b clinical trial of biopsy-confirmed MASH patients with fibrosis (n = 222), 15 mg pegozafermin treatment biweekly for 24 weeks significantly improved fibrosis while resolving MASH (22% of patients with treatment vs. 7% with placebo). These results merited the continued evaluation of pegozafermin in a Phase 3 clinical trial that is actively recruiting MASH patients with fibrosis (NCT06318169). Although the dosing regimens have yet to be determined for this trial, researchers will determine whether pegozafermin treatment causes either fibrosis improvement without worsening MASH or MASH resolution without worsening fibrosis amongst the participants after one year of treatment (n = 1050).

### 2.3. PPAR Agonists

Peroxisome proliferator-activated receptors (PPARs) are ligand-activated nuclear receptors that regulate genes involved in cellular differentiation and metabolic processes, such as lipid and glucose homeostasis [61]. PPARs consist of three major isoforms, PPARα, PPARδ, and PPARγ, which have different tissue distributions, ligand specificities, and physiological functions [28]. Although each of the PPARs has specific natural or synthetic ligands, all isoforms are considered lipid sensors, as they bind to endogenously expressed unsaturated fatty acids that build up in the cell [61]. Upon fatty acid binding, this subgroup of nuclear receptors translocates to the nucleus and heterodimerizes with the retinoid X receptor (RXR). Once in the nucleus, PPARs bind to specific DNA regions, known as peroxisome proliferator hormone response elements (PPREs), to regulate (i.e., transactivate) target gene transcription [28,61]. Upon PPRE binding, PPARα regulates gene transcription specific to lipid catabolism and homeostasis, PPARγ promotes the storage of lipids in adipose tissue and regulates adipocyte differentiation, and PPARδ regulates glucose and lipoprotein metabolism [62]. As a result of their respective regulatory roles, PPARα mainly increases fatty acid oxidation, PPARγ prevents hyperglycemia, and PPARδ increases extrahepatic glucose uptake [61]. Altogether, through this mechanism, PPARs, alongside other nuclear receptors, are crucial regulators of the expression patterns of various metabolic processes that are significant in MASLD/MASH pathology [62].

**Figure 3.**
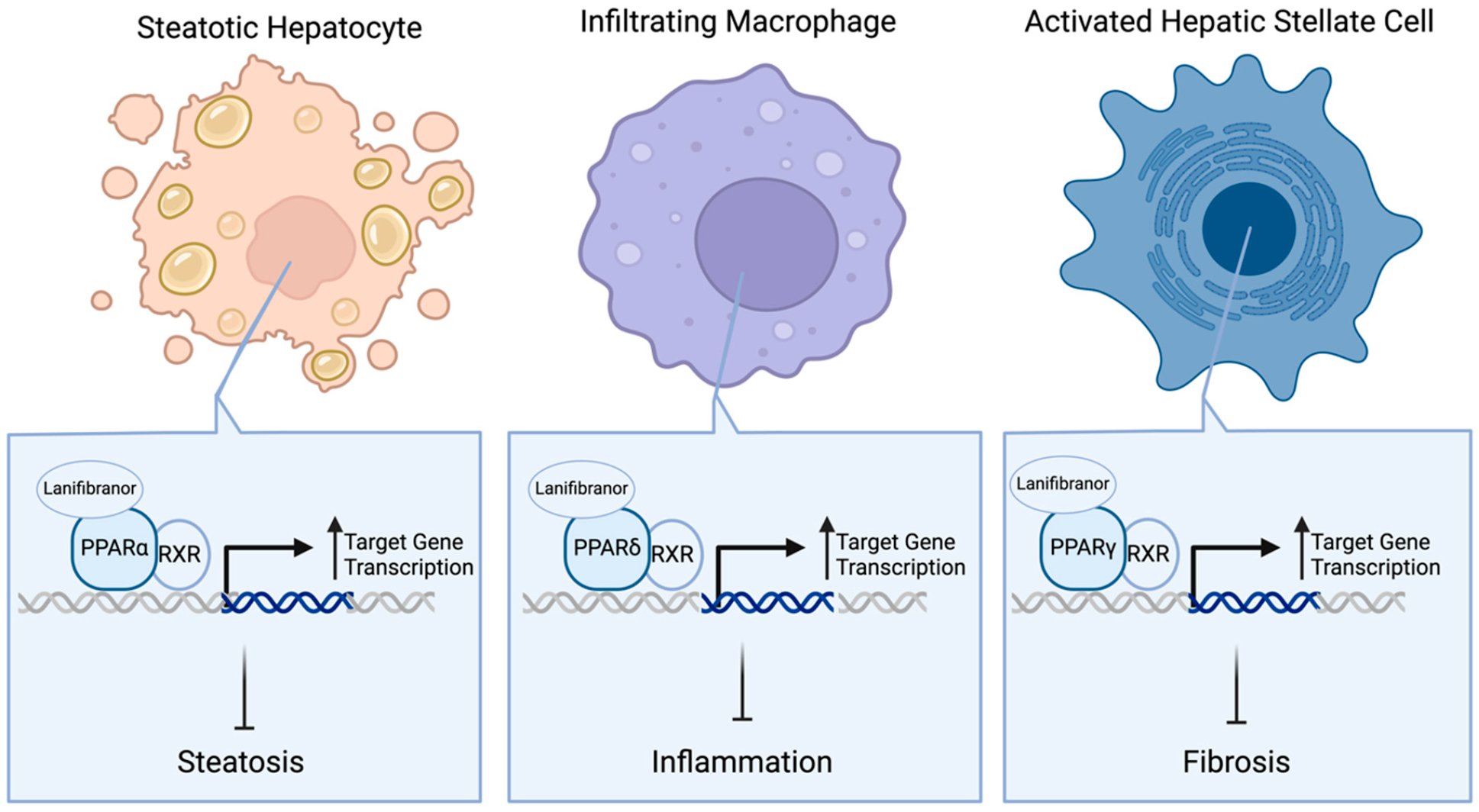
Mechanism of action for pan-PPAR agonists. Upon simultaneous binding of peroxisome proliferator-activated receptor (PPAR) α, δ, and γ, lanifibranor induces the translocation of all three receptors to the nucleus, where they heterodimerize with retinoid X receptor (RXR). Once heterodimerized, all three PPARs bind to their specific peroxisome proliferator hormone response elements (PPREs) to regulate target gene transcription related to steatosis, inflammation or fibrosis. Created in BioRender.

In the clinic, researchers are investigating lanifibranor treatment for MASH patients with T2D (n = 1000) in the NATiV3 clinical trial (NCT04849728). Lanifibranor, a pan-PPAR agonist, directly activates all three PPAR isoforms (Figure 3), resulting in the PPARα-induced inhibition of steatosis in hepatocytes, PPARδ-induced reduction of hepatic macrophage infiltration, and PPARγ-induced reduction of hepatic stellate cell (HSC) activation [63]. By limiting the extent of macrophage infiltration, lanifibranor reduces inflammatory cytokine secretion from these cells that is required to amplify the inflammatory response to injured hepatocytes [64,65]. The attenuation of hepatic stellate cell activation by lanifibranor results in a decreased production and secretion of collagen and fibrogenic cytokines, which are required for the formation of scar tissue and fibrosis [52]. Preclinically, lanifibranor has shown the ability to improve all histological features of steatohepatitis in choline-deficient, amino acid-defined high-fat diet (CDAA-HFD)-fed mice [63]. Furthermore, in the Phase 2b NATiVE trial (NCT03008070), 24 weeks of 800 mg and 1200 mg lanifibranor treatment showed dose-dependent improvement in MASH resolution and fibrosis mitigation by at least 1 stage in MASH patients with liver steatosis (25% of patients with 800 mg and 35% with 1200 mg treatment vs. 9% with placebo; n = 247) [66]. For the NATiV3 trial, patients will receive treatment in two phases. The first phase will be a double-blinded, placebo-controlled administration of 800 mg lanifibranor daily, 1200 mg lanifibranor daily, or a placebo for 72 weeks. MASH resolution and fibrosis improvement will be assessed as defined by the NASH CRN, with at least one stage decrease in fibrosis score as the primary outcome. For the second phase, there will be a double-blinded, placebo-controlled active treatment extension (ATE) up to another 48 weeks, using the same dosing regimen as the first phase, to assess long-term adverse events due to lanifibranor treatment.

### 2.4. SGLT2 Inhibitors

**Figure 4.**
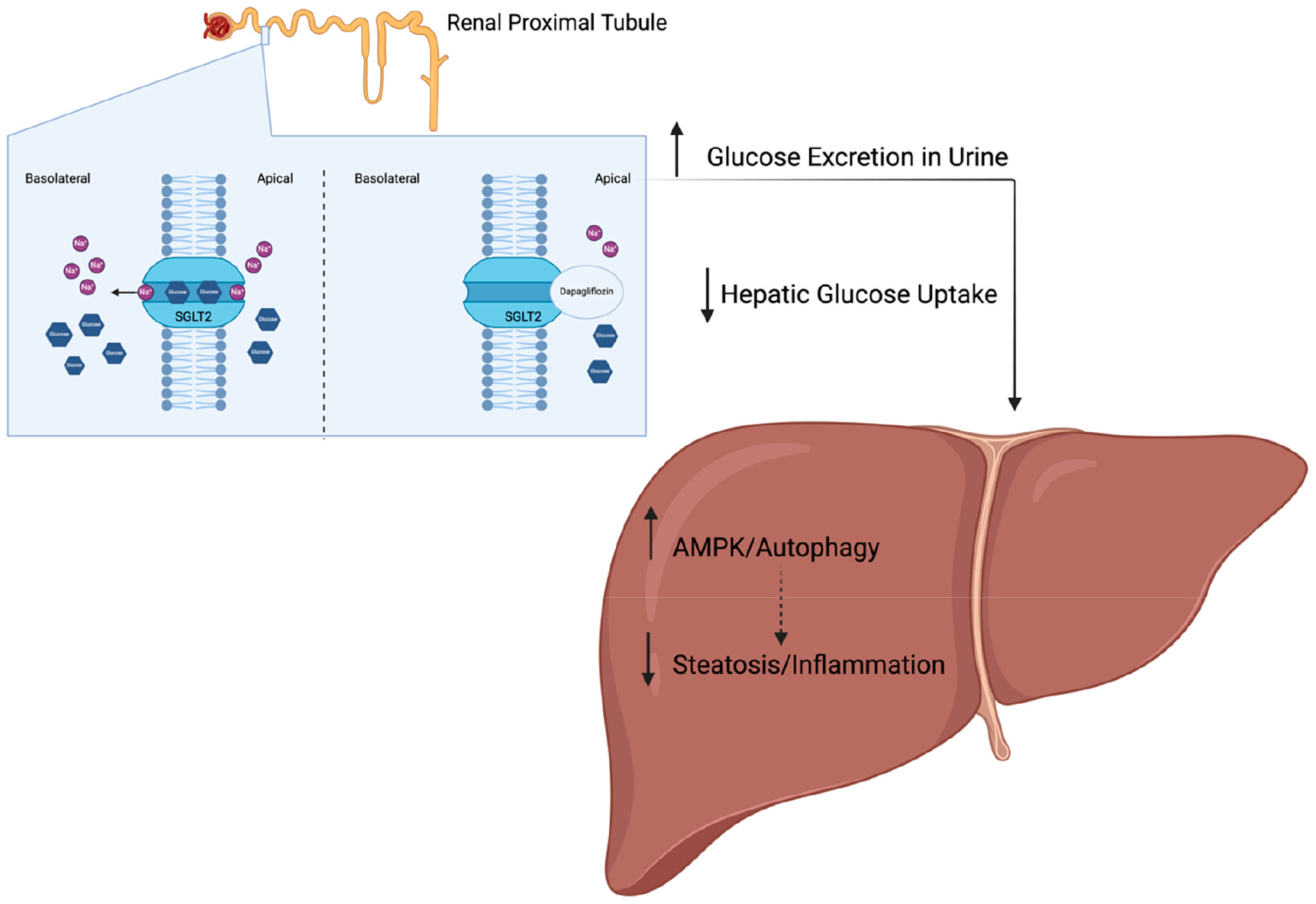
Mechanism of action for SGLT2 inhibitors. Dapagliflozin inhibits sodium–glucose transporter 2 (SGLT2)-mediated sodium (Na^+^) (purple) and glucose (blue) transport, increasing glucose excretion and consequently decreasing hepatic glucose uptake. In result, adenosine monophosphate (AMP)-activated protein kinase (AMPK)-mediated autophagy decreases the extent of steatosis and inflammation in the liver. Created in BioRender.

Sodium–glucose cotransporter 2 (SGLT2) is an ion and metabolite transporter primarily expressed in the proximal tubule of the kidney, responsible for glucose recovery from filtrate [67]. Of particular therapeutic interest are inhibitors of SGLT2, which have been proven to improve glucose control and lower uric acid levels by increasing glucose excretion through the urine as glucose reabsorption in the kidneys via SGLT2 is prevented [26,68]. Due to the reduced glucose reabsorption mediated by SGLT2 in the renal proximal tubule (Figure 4), hepatic glucose uptake is decreased. As a result, adenosine monophosphate-activated protein kinase (AMPK)-mediated autophagy is increased, resulting in certain cellular components of the cytosol being sequestered into vesicles for lysosomal degradation [69–71]. Originally, SGLT2 inhibitors were clinically approved to treat T2D; however, they have recently gained additional approval for use in heart failure and kidney disease patient populations [72–77]. These drugs have additionally received ample attention for expanded use in MASLD/MASH populations. In preclinical models of MASLD, SGLT2 inhibition has been shown to improve lipid metabolism, along with desirable inflammatory responses, reduced oxidative stress, and even apoptosis [78–80]. SGLT2 inhibition has also been shown to increase glucagon secretion, resulting in the stimulation of fatty acid oxidation and consequently reducing liver triglyceride content [26,81–83]. Additionally, SGLT2 inhibition reduces hepatic endoplasmic reticulum (ER) stress, resulting in decreased pro-fibrotic gene expression [26,79,84–86].

Clinically, dapagliflozin has been assessed as a viable therapeutic for MASLD patients, but primarily in the presence of T2D [87]. In a retrospective clinical trial of T2D patients with MASLD, researchers found that dapagliflozin treatment resulted in weight loss (−2.9 kg with treatment vs. −0.4 kg with control; *p* = 0.005) and ALT level normalization (80.0% of patients with treatment vs. 61.5% with control; *p* = 0.041) in concomitant use with metformin (n = 102) [88]. In the “Dapagliflozin Efficacy and Action in NASH” (DEAN) Phase 3 clinical trial, T2D patients with stable glycemic control, defined as HbA1c levels less than 9.5%, that developed MASH within six months of trial start were administered either 10 mg oral dapagliflozin or matching placebo daily for 12 months (n = 154) (NCT03723252). Alteration in scored liver histology was assessed at the end of the treatment period as the primary outcome. Results from this trial have yet to be published.

Interestingly enough, there may be merit in the suggestion that dapagliflozin, along with other SGLT2 inhibitors, may only exhibit ameliorative effects towards MASLD as a result of concomitant metabolic disorders. This is evidenced by the results of a 12-week study in 10 insulin-resistant MASLD patients without T2D, which showed no improvement in hepatic steatosis (12.9% ± 2.9 at baseline vs. 13.2% ± 2.7 at end of study) upon dapagliflozin treatment [89]. Therefore, although promising in its nature, the use of dapagliflozin and other SGLT2 inhibitors requires fine-tuning for MASLD/MASH efficacy, with respect to experimental design and patient characteristics.

### 2.5. PDE Inhibitors

Phosphodiesterase enzymes (PDEs) have been implicated in upregulating inflammatory responses, such as increased oxidative stress as well as tumor necrosis factor (TNF)-α and cytokine production, making the inhibition of this enzyme a desirable therapeutic strategy [31,90]. Additionally, PDEs have been implicated in obesity and T2D, as evidenced by a PDE4 knockout mouse model with reduced adiposity and the associated inflammation, alongside a separate mouse model treated with a PDE4 inhibitor that improved glucose tolerance and insulin sensitivity [91,92]. As a result, PDE inhibitors, such as the hemorheological agent pentoxifylline (PTX), have been of particular interest in their potential to treat MASLD/MASH [93]. Through the non-selective inhibition of PDEs on the plasma membrane of HSCs (Figure 2), PTX increases cyclic AMP levels in the cytoplasm to subsequently activate protein kinase A (PKA) [94,95]. The downstream nuclear factor kappa B (NF-κB) and transforming growth factor (TGF)-β signaling activities are then inhibited, resulting in decreased inflammation and fibrosis, respectively [96–98].

Clinically, there have been conflicting outcomes associated with PTX treatment for MASLD/MASH. In a previous clinical trial comparing PTX efficacy in MASH improvement against a placebo (n = 55), 400 mg PTX administration three times per day was found to significantly reduce steatosis (−0.9 mean change in NAS score vs. 0.04 with placebo; *p* < 0.001), lobular inflammation (−1 median change in NAS score vs. 0 with placebo; *p* = 0.02), and liver fibrosis (−0.2 mean change in fibrosis score vs. +0.4 with placebo; *p* = 0.038) without any significant adverse events [99]. However, a later trial of MASH patients (n = 26) proved PTX’s ability to improve liver function to be inconsistent, as liver enzyme levels, along with other biochemical measures, did not significantly differ from the results of the placebo group (*p* = 0.48) [100]. In a recently completed Phase 3 trial, MASH patients (n = 50) received the same treatment regimen with standard care and were assessed for liver function along with NAFLD fibrosis score (NFS) after a six-month treatment (NCT05284448). However, results from this trial have yet to be made public

### 2.6. AMPK Activators

AMPK, a heterotrimeric serine/threonine (Ser/Thr) protein kinase complex, is widely regarded as a major sensor of cellular energy status in eukaryotes [101]. Upon cellular stress conditions, such as hypoxia and glucose deprivation, AMPK is activated to simultaneously inhibit ATP consumption by anabolic processes, such as protein and fatty acid synthesis, while enhancing ATP synthesis by catabolic processes, such as fatty acid oxidation. It has been widely known that AMPK activation attenuates hepatic steatosis by reducing hepatic lipid stores [102]. AMPK activation has also been shown to reduce the expression of pro-inflammatory mediators, consequently attenuating inflammation in various contexts [102–104]. Additionally, AMPK activation has been shown to not only reduce apoptosis by decreasing caspase signaling, but also inhibit HSC activation and TGF-β signaling, both of which are required for fibrosis progression [102,105,106].

**Figure 5.**
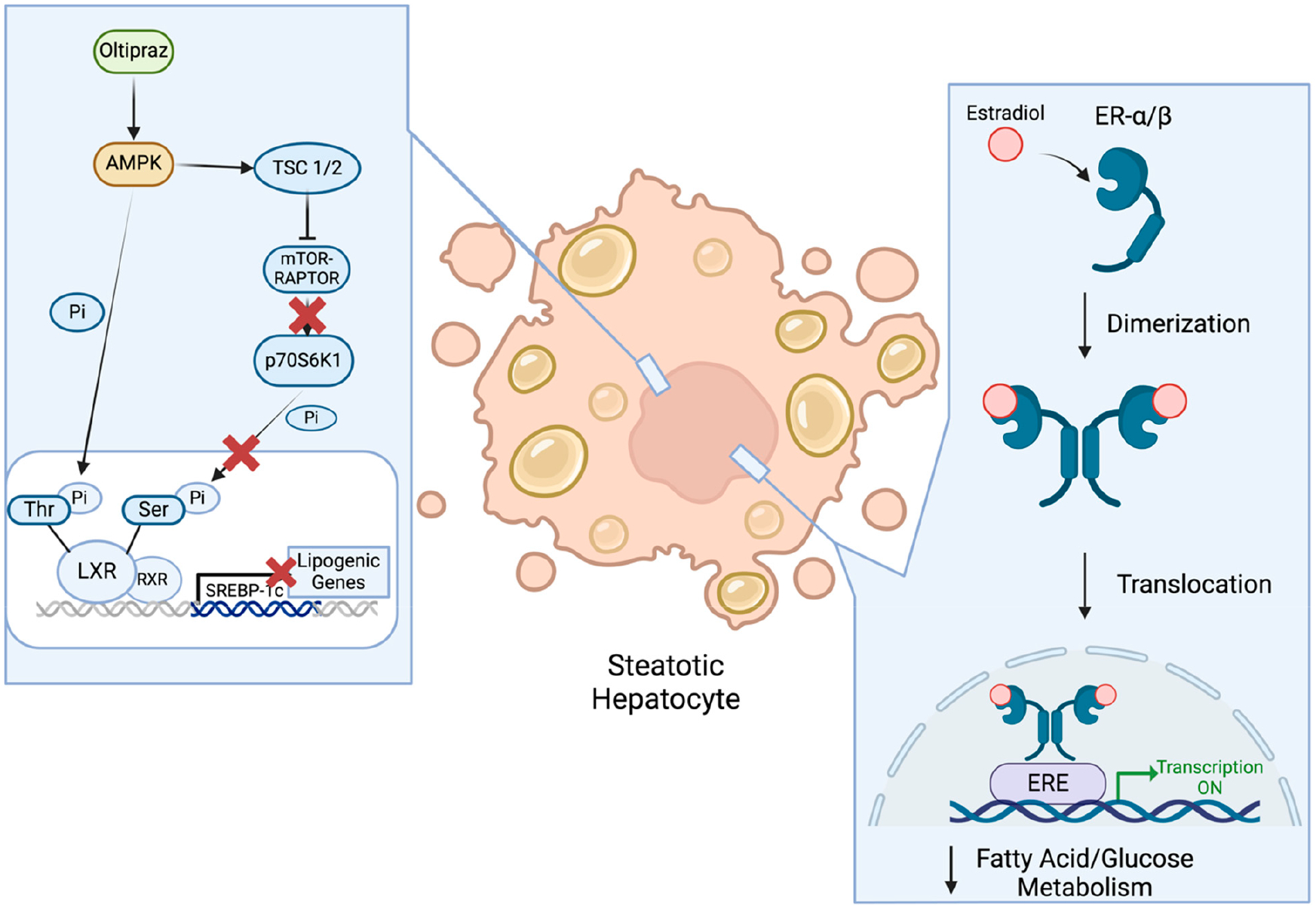
Potential MASH therapeutics primarily targeting nuclear receptors. By activating AMPK, Oltipraz inhibits p70 ribosomal S6 kinase-1 (S6K1)-mediated liver X receptor (LXR) activation, which is required for sterol regulatory element-binding protein-1c (SREBP1-c-mediated) gene transcription, reducing lipogenic expression required for fatty acid synthesis. Estradiol binds and induces conformational changes in estrogen receptor (ER) α/β to promote the receptors’ nuclear translocation. Upon this translocation, estrogen response elements (EREs) bind to the receptors to promote target gene transcription, subsequently lowering cholesterol levels and fat accumulation in the liver. Refer to the text for further mechanistic details. Refer to Figure 3 for the mechanism behind therapeutics targeting PPAR nuclear receptors. Created in BioRender.

Recently, Oltipraz, an AMPK activator (Figure 5), has been evaluated in MASLD patients with no indications of cirrhosis for 24 weeks (n = 146) to assess its effect on liver fat deposition by using magnetic resonance spectroscopy (MRS) (NCT04142749). Oltipraz has been found to prevent insulin resistance via the inhibition of p70 ribosomal S6 kinase-1 (S6K1). By stimulating AMPK activation, Oltipraz indirectly activates tumor-suppressor complex 1/2 (TSC1/2) to inhibit mTOR-Raptor complex activation [29]. Therefore, S6K1 remains inactive and is unable to phosphorylate nuclear receptor liver X receptor-α (LXRα) on Ser residues of LXRα, in favor of the AMPK phosphorylation of LXRα on Thr residues to keep the nuclear receptor inactive. As a result, the S6K1-mediated activation of LXRα is inhibited, preventing sterol regulatory element-binding protein-1c (SREBP-1c)-mediated gene transcription for fatty acid synthesis [29]. Nevertheless, results from this trial have yet to become public, even though its completion was almost two years ago, leaving one to wonder if targeting AMPK alone is sufficient to treat MASH.

### 2.7. Voltage-Gated Chloride Channel Activators

Chloride channels (ClCs) have become of interest lately in the field of MASLD/MASH drug development. ClCs are ion transporters that act as either electrodiffusive chloride (Cl^−^) channels or secondary active transporters in exchange for protons (H^+^) [107]. Both classes of ClCs contain a conserved glutamate (Glu) residue responsible for Cl^−^ and H^+^ exchange [108]. Mechanistically, ClCs, such as ClC-1, are voltage-gated channels that are in an open conformation at 0 mV and closed upon hyperpolarization, the application of a negatively charged transmembrane voltage [107]. In contrast, ClC-2 opens upon hyperpolarization and rests in the closed state at 0 mV [109,110].

Although ClC-2 is ubiquitously expressed in the body, previous work has been focused on its pathophysiological role in cardiovascular and ocular impairments caused by its deficiencies [109,111,112]. A particular drug of interest is lubiprostone, a ClC-2 activator that causes Cl^−^ efflux into the gastrointestinal (GI) lumen, promoting intestinal fluid secretion [113]. Although a direct mechanism of action for this compound in regard to the development of MASLD/MASH has yet to be characterized, it is believed that lubiprostone reduces liver injury via the gut–liver axis [114]. The gut–liver axis is characterized by the reciprocal relationship between the gut and the liver based upon signaling activities induced by dietary, genetic, and environmental factors [114]. Previous research indicates that diets high in fat alter the gut microbiome in a manner that impairs both the intestinal barrier and the gut vascular barrier. As a result, this disruption mediates the influx of bacterial products from the gut into the hepatic portal vein, consequently inducing both inflammation and metabolic abnormalities [114,115]. This phenomenon suggests that, by promoting intestinal fluid secretion, lubiprostone prevents gut endotoxins from leaking out of the gut and into the bloodstream to be absorbed by the liver. As a result, the uptake of gut endotoxins into the liver decreases, reducing the extent of endotoxemia-induced hepatic injury [30,116]. In a Phase 2a study in MASLD patients experiencing constipation (n = 150), lubiprostone was found to reduce the patients’ liver enzyme levels (−13 ± 19 U/L with treatment vs. 1 ± 24 U/L with placebo; *p* < 0.0007) [117]. However, a caveat to this clinical trial is that roughly 30 percent of patients receiving the higher dosage of lubiprostone began to develop side effects such as diarrhea, vomiting, and nausea. More recently, a Phase 3 trial assessing lubiprostone’s ability to decrease liver fat content, by using MRI-PDFF, in MASLD patients (n = 116) has completed its 48-week treatment regimen (NCT05768334). However, the results from this trial have yet to be published.

### 2.8. GSH Precursors A

Glutathione (GSH) is a water-soluble, ubiquitously present thiol antioxidant composed of the tripeptide cysteine, glycine, and glutamate [32]. Cysteine is of particular importance due to its thiol group providing GSH with antioxidant properties in the body. The precursors of GSH, especially N-Acetyl Cysteine (NAC), have become major topics of discussion as not only a dietary supplement but also a drug candidate.

NAC is commonly referred to in the literature as “dietary GSH”, serving as the xenobiotic precursor to GSH, which is capable of reducing oxidative stress [32]. Oxidative stress is a result of an imbalance between reactive oxygen species (ROS) (i.e. free radicals) generation and antioxidants, in which there are insufficient amounts of antioxidants to donate electrons to ROS to reduce their damage to the cell [118,119]. ROS oxidize phospholipids on the cellular membrane, producing an apoptotic chain reaction of free radicals, inducing inflammation and fibrosis [119–121]. Through the induction of hepatocyte apoptosis, ROS stimulates the release of damage-associated molecular patterns (DAMPs) capable of both activating Kupffer cells and recruiting additional immune cells [121]. Consequently, cytokine and chemokine production are amplified, resulting in the subsequent activation of HSCs required for the production of extracellular matrix (ECM) proteins and eventual fibrosis. Recently, a Phase 3 trial of MASLD patients was completed, where participants underwent a weight reduction program with or without 2400 mg NAC per day for 3 months (n = 60) (NCT05589584). Notably, the plasma level of leptin, an insulin resistance marker for MASLD, was utilized as the primary outcome in this trial. However, the results from this trial have yet to be made publicly available. Clinically, although promising data have suggested NAC’s efficacy in T2D and MASLD, there are regulatory issues related to the safety of the compound [32].

### 2.9. Estrogens

Estrogen is a collection of female sex hormones that can bind to nuclear and membrane receptors [33]. When binding to either estrogen receptor (ER)-α or -β, estrogens stimulate conformational changes in the receptors to form homo- or heterodimers. Upon these conformational changes, estrogen response elements (EREs) in target gene regulatory regions are bound to the receptors, promoting target gene expression to counteract fatty acid and glucose metabolic processes (Figure 5). Although more commonly synthesized by the gonads, estrogens have also been shown to be synthesized in the liver and adipose tissues [33,122]. In both males and females, estrogen signaling controls glucose and cholesterol homeostasis along with lipogenesis [33]. By regulating glucose homeostasis and insulin clearance, estrogens decrease gluconeogenesis while simultaneously increasing hepatic glycogen synthesis and storage, thereby regulating plasma levels of glucose [123–125]. Low levels of estrogens in post-menopausal women have been implicated with elevated levels of plasma cholesterol and low-density lipoproteins (LDLs), promoting fat accumulation while, simultaneously, altering lipid homeostasis in the liver.

In the clinic, hormonal replacement therapy has been of high interest in treating MASLD [33]. In an actively recruiting Phase 3 trial (NCT04833140), post-menopausal female patients with an intact uterus (n = 60) will receive a 100 μg estradiol patch daily, along with a 100 mg progesterone vaginal tablet every day to prevent the development of endometriosis [126]. After a treatment period of one year, researchers will assess any changes in liver fibrosis and fat content as primary outcomes, but the measures of assessing these outcomes have not been released. Hormonal replacement therapies for MASLD/MASH still require a lot of progress before clinical approval, however. Evidence has arisen that they increase the likelihood of breast cancer, dementia, blood clotting, and stroke development when used long-term [127]. All in all, estrogen replacement therapies still need to be fine-tuned to ensure safety in the female population.

### 2.10. Galectin-3 Inhibitors

**Figure 6.**
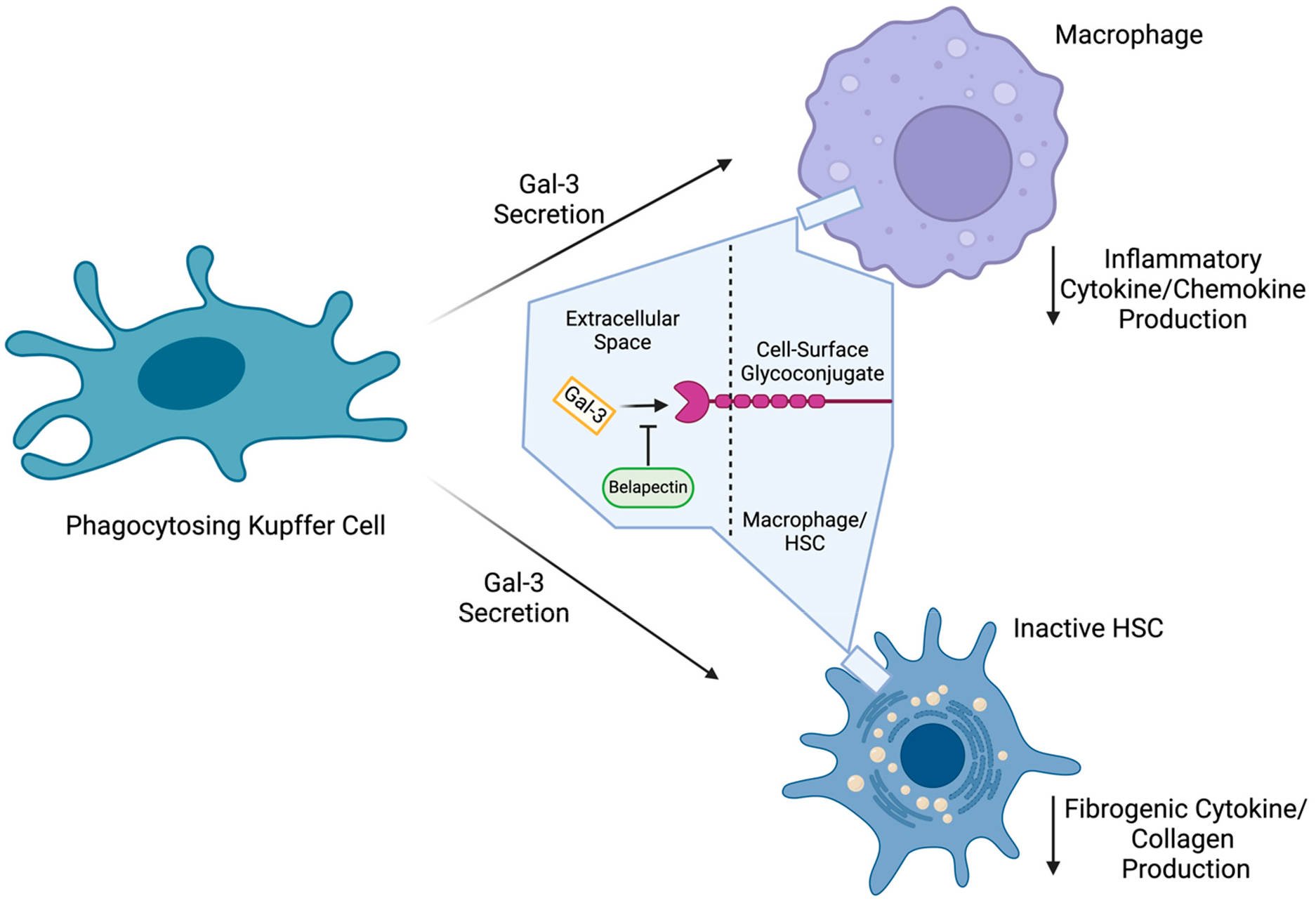
Mechanism of action for galectin-3 inhibitors. Belapectin directly binds extracellular galectin (Gal)-3 secreted from phagocytosing Kupffer cells, antagonizing Gal-3-mediated macrophage and hepatic stellate cell (HSC) activation. As a result, belapectin dampens inflammatory and fibrotic responses mediated by these cell types. Created in BioRender.

Galectins are carbohydrate-binding proteins with pronounced expression in states of inflammation, fibrosis, and cancer in several organs in the body (e.g., heart, kidney, and liver) [128,129]. Of particular interest is galectin-3 (Gal-3), which is highly expressed by macrophages in disease states relevant to hepatic fibrosis [130,131]. In a mouse model of Gal-3 deficiency, the mice were protected from diet-induced MASH [132]. Additionally, the results from the knockout mice suggest that Gal-3 promoted both inflammation and fibrosis, which was further corroborated by the findings from high-fat-diet-fed mice, uncovering that Gal-3 inhibition reduces hepatic lipid accumulation [132–135]. Most notably, a transcriptomics analysis in pigs found that Gal-3 expression was significantly associated with the steatosis–steatohepatitis transition, suggesting the potential use of Gal-3 expression as a biomarker of the progression and reversal between steatosis and steatohepatitis [136]. Because of these findings, inhibitors of Gal-3 (Figure 6), such as belapectin, have been a major point of interest in the search for MASH therapeutics. By binding to Gal-3 upon secretion from Kupffer cells, belapectin prevents Gal-3 from binding to cell surface receptors on macrophages to initiate inflammatory processes, as well as binding and activating HSCs, which play a critical role in fibrotic processes [129,133,137–139]. In a diet-induced mouse MASH model, belapectin was shown to reduce Gal-3 expression in liver macrophages, reduce hepatocyte fat accumulation and ballooning, and reduce fibrosis as measured by liver collagen levels [34].

Clinically, however, belapectin has not achieved the same extent of success, as evidenced by a recent Phase 2b trial of biweekly belapectin treatment for one year [140]. In MASH patients with cirrhosis and portal hypertension (n = 162), belapectin treatment was unable to significantly reduce fibrosis or the hepatic venous pressure gradient (HPVG), a measure of portal hypertension, as compared to the placebo. However, the researchers did notice that belapectin had efficacy in a subgroup of MASH patients without esophageal varices (n = 81), providing merit in continuing with a Phase 2b trial in such MASH patients (NCT04365868). In this trial, patients will be treated biweekly with either 2 or 4 mg/kg lean body mass belapectin through IV injection for 78 weeks (n = 357) and then be assessed by the instance of new esophageal varices formation, which is considered an early sign of complications related to cirrhosis [141]. Once results are collected and the optimal dose is determined, this trial will then move to Phase 3 to repeat the design in the same patient population.

## 3. Suspended/Terminated Candidate Therapeutics for MASH

As seen from the vastly different pathways targeted by the candidates above, the researchers and pharmaceutical industry have been searching high and low for an answer to the MASH problem. However, just as there are numerous candidates being swiftly evaluated as viable solutions, there have been plenty of therapeutics that have not had the same luck, whether it be poor efficacy, safety concerns, or sponsors pivoting away from the proposed compound. With that being said, understanding the mechanistic background and liver pathology related to these compounds is crucial in furthering the development of MASH therapeutics.

### 3.1. SCD1 Inhibitors

Aramchol is an inhibitor of hepatic stearoyl-CoA desaturase 1 (SCD1), preventing the conversion of saturated fatty acids into monounsaturated fatty acids [142,143]. As a result, aramchol treatment has been shown to elevate PPAR-γ levels while reducing the levels of fibrogenic factors [144]. In a global *SCD1* knockout mouse model, SCD1 deficiency was found to cause an increased hepatic gene expression of fatty acid oxidation while simultaneously decreasing hepatic triglyceride synthesis and its secretion from the liver [145]. The efficacy of aramchol against MASH was preliminarily investigated in a Phase 3 clinical trial (NCT04104321). The trial planned to assess a twice daily administration of 300 mg aramchol in MASH patients with liver fibrosis in an open-label fashion for 72 weeks (n = 2000). The primary outcomes revolved around safety, pharmacokinetics (PK), liver histology, and non-invasive tests (NITs) associated with MASH and fibrosis at 24, 48, and 72 weeks of treatment. Pending the results of the open-label study, a double-blinded trial might be conducted, according to the released study design. However, due to the positive findings in the open-label study showing significant fibrosis improvement by aramchol treatment, based on NASH CRN and digital pathology measurements [146], the double-blinded trial has been put in suspension, as the sponsors are re-evaluating their clinical strategy, along with pivoting to a different formulation of the compound, from aramchol free acid to aramchol meglumine [147].

### 3.2. Antioxidants

Metadoxine is an antioxidant which has been shown to restore the levels of nicotinamide adenine dinucleotide (NADH), glutathione (GSH), and adenosine triphosphate (ATP), along with the proportion of saturated and unsaturated fatty acids and esters in the liver [148,149]. Metadoxine has also been shown to decrease the synthesis of fibronectin and procollagen, along with preventing collagen deposition and TNF-α secretion caused by acetaldehyde in HSCs [150]. Clinically, although the twice daily administration of 500 mg metadoxine did improve ultrasound-assessed steatosis in MASH patients (n = 134), the agent had no impact on liver histology or serum ALT/AST levels [149]. More recently, the efficacy of metadoxine was investigated in a Phase 3 trial for MASH patients, who were overweight or obese, without cirrhosis (NCT02541045). These patients would be administered either 500 mg metadoxine twice per day or a matching placebo for six months. However, due to a lack of resources to fund the trial, the sponsor suspended the trial.

### 3.3. FXR Agonists

OCA, a farnesoid receptor agonist (FXR), has been shown to regulate the hepatic metabolism of bile and cholesterol by binding to FXR in hepatocytes and intestinal cells [151,152]. Upon ligand binding, FXR translocates into the nucleus and heterodimerizes with RXR to induce the transcription of proteins such as small heterodimer protein (SHP) [153,154]. Through SHP-mediated inhibition of CYP7A1, the cytochrome p450 enzyme responsible for the conversion of cholesterol into bile acids, bile production is suppressed, subsequentially decreasing hepatocyte exposure to excess bile and increasing liver bile flow [151,152,155]. Over the years, there has been solid evidence from preclinical and clinical findings supporting the efficacy of OCA and other FXR agonists against MASH [6]. However, the tides have seemed to turn against OCA based upon the results of the recent “Randomized Global Phase 3 Study to Evaluate the Impact on NASH with Fibrosis of Obeticholic Acid Treatment” (REGENERATE) trial (NCT02548351). In the REGENERATE trial, although the sponsor reported encouraging data regarding the efficacy of 10 or 25 mg daily treatment in MASH patients (n = 2477) [156], the FDA rejected the sponsor’s New Drug Application (NDA), citing safety concerns with OCA treatment resulting in druginduced liver and kidney injuries [157]. In addition, the FDA questioned the validity of the findings regarding fibrosis, as the sponsor utilized non-invasive techniques to assess fibrosis improvement, in lieu of biopsy.

### 3.4. CCR 2/5 Antagonists

Cenicriviroc (CVC), a dual C-C motif chemokine receptor (CCR) 2/5 antagonist, received some attention as a candidate for MASH treatment a few years ago. Through CCR2/5 antagonism, CVC prevents C-C motif ligand 2 (CCL2) and CCL5 binding to their respective receptors, reducing monocyte infiltration and altering hepatic macrophage subsets to alleviate fibrosis [158]. Because of this, CVC was regarded as a plausible candidate for MASH treatment and assessed in a Phase 3 clinical trial back in 2017 (NCT03028740). In this study, MASH patients (n = 1778) received 150 mg CVC treatment daily for 12 months to assess its effects on fibrosis and steatohepatitis via NASH CRN scores. Depending upon the results of the 12-month treatment regimen, the trial was designed with a 42-month treatment period to assess long-term side effects from treatment. However, CVC was unable to achieve efficacy in alleviating fibrosis as compared to the placebo, resulting in the trial’s termination [159].

### 3.5. ASK1 Inhibitors

Selonsertib is an inhibitor of apoptosis signal-regulating kinase 1 (ASK1). It binds to ASK1’s catalytic kinase domain in an ATP-dependent manner [160]. Through this binding, selonsertib inhibits ASK1’s ability to phosphorylate and activate downstream proteins—mitogen-activated protein kinase kinase 3/6 (MKK3/6) and p38-mitogen-activated protein kinase (MAPK)—which promote fibrosis and inflammation [161]. In preclinical settings, selonsertib has been shown to suppress HSC growth and proliferation, and even alleviate dimethylnitrosamine (DMN)-induced liver fibrosis in rats [162]. However, the preclinical efficacy of selonsertib has not been translated in the clinic. Recently, a Phase 3 trial was conducted to determine if 18 mg selonsertib treatment for 48 weeks could ameliorate fibrosis without worsening MASH in MASH patients with bridging fibrosis (n = 808) (NCT03053050). The researchers originally planned to follow up this trial with a long-term study to assess if there were any long-term side effects; however, the first phase of the trial proved to be unsuccessful [163]. Although selonsertib treatment lacked any signs of significant adverse events, it was unable to alleviate either fibrosis or MASH progression in comparison to the placebo, resulting in the trial’s termination.

## 4. Concluding Remarks

As one can see, the pharmaceutical world has been leaving no stone unturned to find the best possible therapeutic agent to treat MASH. One sponsor went as far as to determine if a plaque psoriasis monoclonal antibody could be used for MASH, based primarily on a meta-analysis correlation [164,165]. GLP-1R agonists and FGF21 analogues seem to be very promising to join the likes of Resmetirom for FDA approval as MASH therapeutics in the US. Furthermore, there are various potential therapeutics and targets additionally being investigated for MASH treatment that have not reached late-phase clinical trials per the publishing of this review. For example, a more novel target being investigated involves the modulation of neutrophil extracellular traps (NETs). This immune cell-mediated antimicrobial mechanism has shown to be positively correlated to MASH development, as excess NETosis, the process of neutrophils releasing extracellular traps, may induce autoimmunity, endothelial damage, and inflammation [166–170]. Considering the potential role of Angiotensin II (Ang II), a well-known target for hypertension treatment, in the induction of NETosis and its associated fibrotic damage, the Ang II receptor blocker (ARB) Telmisartan has undergone small-scale clinical trials to assess its utility in MASH treatment [171,172]. Nevertheless, due to the differing targets of each therapeutic and their downstream effects in the liver, determining whether one therapeutic is a “better” treatment than the other for MASH is still comparing apples to oranges.

Based upon the vast number of mechanisms being targeted by sponsors, MASH seems to require a treatment that manages to target more than one particular network of inflammation, fibrosis, and/or lipid accumulation. Researchers have been catching onto the trend of targeting MASH in a multifaceted manner and have begun looking into combined regimens of therapeutics to see if modulating multiple targets could be more efficacious in defeating MASH [173]. For example, although sponsors for OCA have been struggling to obtain their drug’s approval as a stand-alone MASH treatment, OCA’s combined effectiveness with elafibranor, a dual PPARα/γ agonist, has been assessed in preclinical settings [174]. Alternatively, although there was no update regarding any approval of aramchol as a stand-alone MASH therapeutic, the sponsors have been recently granted patent approval for the combined use of aramchol with Resmetirom for MASH patients [175]. Most notably, Gilead Sciences has been investigating the combined efficacy of two new drugs, with different mechanisms of action, alongside semaglutide in clinical trials (NCT03987074). Cilofexor, a FXR agonist, in combination with firsocostat, an acetyl-CoA carboxylase (ACC) inhibitor in the AMPK pathway, and/or semaglutide has been assessed in MASH patients (n = 109) for 24 weeks in a Phase 2a trial. The results have indicated that the combinatorial treatment was not only safe, but further alleviated liver steatosis, as measured by MRI-PDFF, liver enzyme levels, and non-invasive measures of fibrosis [176]. It should be noted, however, that although it is theoretically ideal to account for the systemic pathology of MASH, pursuing combinatorial regimens for MASH treatment contains regulatory roadblocks elongating the development and investigation of the proposed therapy. According to FDA guidance on combination therapy development, sponsors must provide proof of each monotherapy achieving efficacy that is further improved when administered with the other proposed therapeutics [173]. In addition, there must be evidence of each monotherapy’s contribution toward the efficacy of combined treatment, whether it be increasing treatment potency, improving clinical efficacy, or reducing the incidence and severity of side effects [173].

Additional challenges beyond the scope of drug development may hamper our efforts to develop MASH therapeutics. Recently, a vast panel of experts from various personal and professional backgrounds laid out a comprehensive action plan, bringing to light and addressing issues which have been holding back the development of treatment options for MASLD and MASH [177]. In this agenda, they propose avenues for advancing awareness and education about the disease for patients and medical providers, implementing novel methods of primary care, and even calling for changes in public health policy to create a global strategy for the treatment and prevention of MASLD and MASH.

While MASH’s impact has been felt amongst various populations for decades now, FDA-approved treatments for this “silent” disease are slowly but surely coming to the rescue. Hopefully, the recent success of Resmetirom is a sign of more to come, with other therapeutics receiving regulatory approval and opening the gates to eradicating this disorder.

## Figures and Tables

**Table 1.** Potential MASH therapeutics undergoing investigation in late-phase clinical trials. See cited references under “Drug Class” for more information. GLP-1, glucagon-like peptide-1; FGF21, fibroblast growth factor 21; PPAR, peroxisome proliferator-activated receptor; SGLT2, sodium–glucose cotransporter 2; AMPK, adenosine monophosphate-activated protein kinase; ClC-2, chloride channel-2; ER, estrogen receptor; Gal-3, galectin-3.

Drug Candidate	Drug Class	Trial Phase	Trial Status	Clinical Trial Registration	Primary Endpoint
Semaglutide	GLP-1 Agonist [26]	3	Active	NCT04822181	Part 1 (After 72 Weeks): Resolution of Steatohepatitis and No Worsening of Fibrosis Improvement in Liver Fibrosis and No Worsening of Steatohepatitis Part 2 (After 240 Weeks): Cirrhosis-Free Survival
Survodutide	GLP-1 Agonist [26]	3	Recruiting	NCT06309992 NCT06632444	NCT06309992: After 48 Weeks: Relative Reduction in Liver Fat Content ≥30% Relative % Change in Body Weight NCT06632444: Part 1 (After 52 Weeks): MASH Resolution Without Worsening Fibrosis ≥1-Point Improvement in Fibrosis Stage Without Worsening MASH Part 2 (Up to 7 Years): Incidence of Liver-Related Adverse Event/All-Cause Mortality
Efruxifermin	FGF21 Analogue [27]	3	Recruiting	NCT06161571 NCT06215716	NCT06161571: After 52 Weeks: Time/Incidence/Severity of Adverse Events Incidence of Clinically Significant Changes NCT06215716: After 52 Weeks: Resolution of MASH and ≥1 Stage Fibrosis Improvement After 240 Weeks: Event-Free Survival
Pegozafermin	FGF21 Analogue [27]	3	Recruiting	NCT06318169	After 52 Weeks: Incidence of Fibrosis Improvement ≥ 1 Stage Without Worsening of MASH Incidence of MASH Resolution Without Worsening of Fibrosis
Lanifibranor	Pan-PPAR Agonist [28]	3	Recruiting	NCT04849728	Part A (After 72 Weeks): Resolution of MASH/Fibrosis Improvement Part B (After 48 Weeks Post-Part A Completion): Incidence of Adverse Events
Dapagliflozin	SGLT2 Inhibitor [26]	3	Completed (Results Pending)	NCT03723252	After 12 Months: Scored Liver Histological Improvement
Oltipraz	AMPK Activator [29]	3	Completed (Results Pending)	NCT04142749	After 24 Weeks: Change in Assessed Liver Fat
Lubiprostone	ClC-2 Activator [30]	3	Completed (Results Pending)	NCT05768334	After 48 Weeks: Change in Fat Quantification
Pentoxifylline (PTX)	PDE Inhibitor [31]	3	Completed (Results Pending)	NCT05284448	After 6 Months: Improvement in Liver Aminotransferases (ALT and AST) Change in NAFLD Fibrosis Score (NFS)
N-Acetyl Cysteine (NAC)	Antioxidant [32]	3	Completed (Results Pending)	NCT05589584	After 3 Months: Assessment of Leptin as Measure of Insulin Resistance
Estradiol	ER Agonist [33]	3	Recruiting	NCT04833140	After 12 Months: Reduction of Liver Fibrosis Reduction in Liver Fat
Belapectin	Gal-3 Inhibitor [34]	2b	Active	NCT04365868	After 78 Weeks: Incidence of Newly Formed Esophageal Varices
